# A Challenging Case of Ileosigmoid Knotting in an Elderly

**DOI:** 10.7759/cureus.9624

**Published:** 2020-08-09

**Authors:** Pradeep Kumar Singh, Manwar S Ali, Das B Manohar, Mahesh Sethi

**Affiliations:** 1 General Surgery, All India Institute of Medical Sciences, Bhubaneswar, IND

**Keywords:** ileosigmoid knotting, isk, elderly, double stoma

## Abstract

Ileosigmoid knotting (ISK) is one of the rare causes of acute intestinal obstruction, which has a rapid course for forming gangrene. ISK is considered a variant of sigmoid volvulus, which otherwise is called compound volvulus. The difficulty in ISK diagnoses is owing to its rarity, uncommon presentation, and non-specific radiological findings. The physiological status of the patient and intraoperative findings are the key factors in deciding the operative procedure of choice. Herein the author describes a rare case of ISK in a 70-year-old man with gangrenous ileum and sigmoid colon, which were treated successfully with resection and double stoma.

## Introduction

Ileosigmoid knotting (ISK) is a rare condition to encounter at an emergency doorstep, where the ileal loop twists around the sigmoid colon and its mesentery or the opposite way. It is more common in males, with a mean age of 40 years. It culminates in double-loop obstruction, which can be gangrenous or non-gangrenous at presentation. Effective resuscitation, prompt diagnosis, and early surgical intervention are the key factors in deciding postoperative mortality and morbidity. Surgical management of ISK is controversial and is based on the patient’s hemodynamic status and intraoperative findings. The primary aim of surgery is to untwist the knot, resect any gangrenous segment, and restore bowel continuity. However, in patients with unstable vital signs, advanced age, comorbidities, or peritonitis, or in whom anastomosis is difficult and precarious, resection with stoma may be necessary [1].

## Case presentation

**Figure 1:**
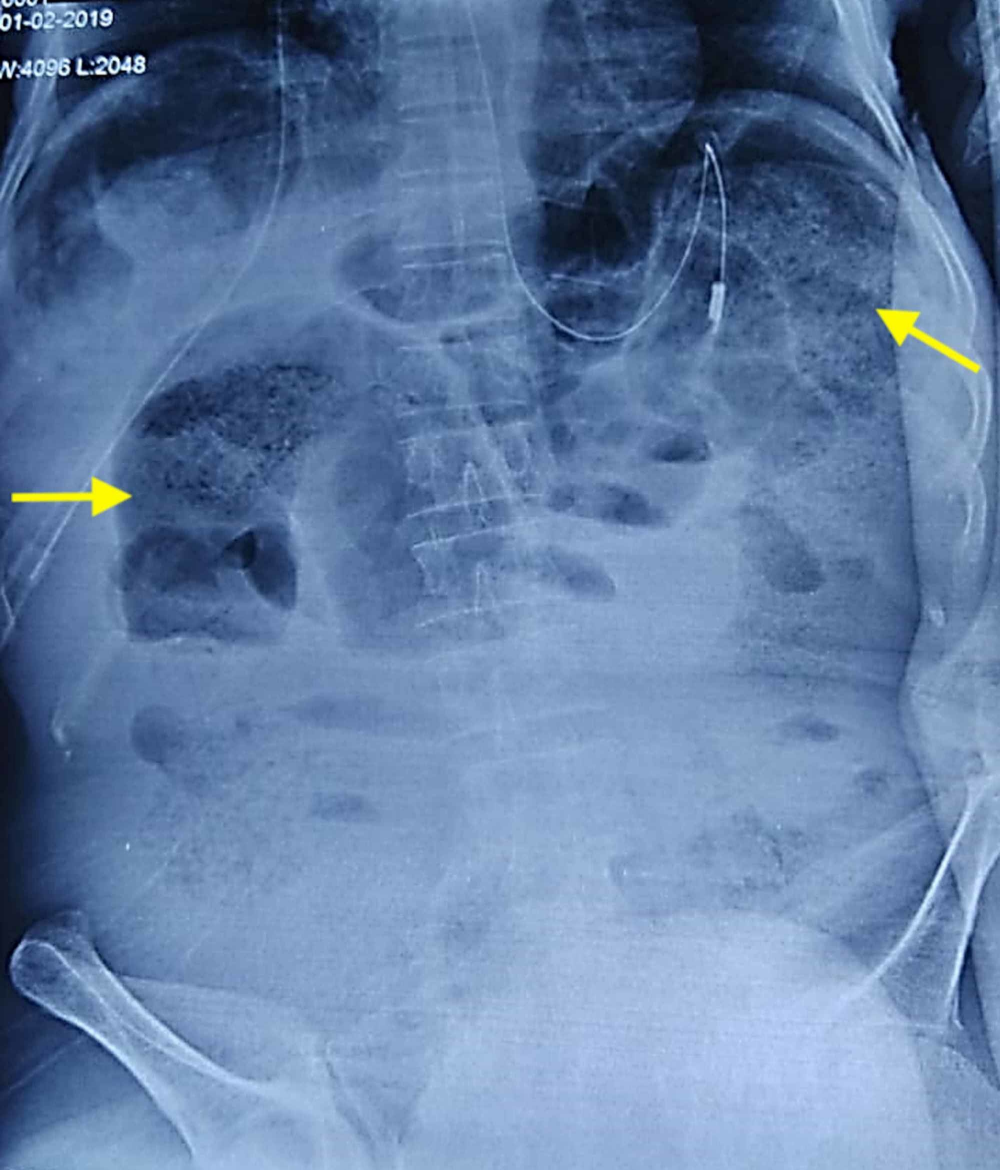
Preoperative X-ray of the abdomen, with the yellow arrows showing the distended bowel loops.

**Figure 2:**
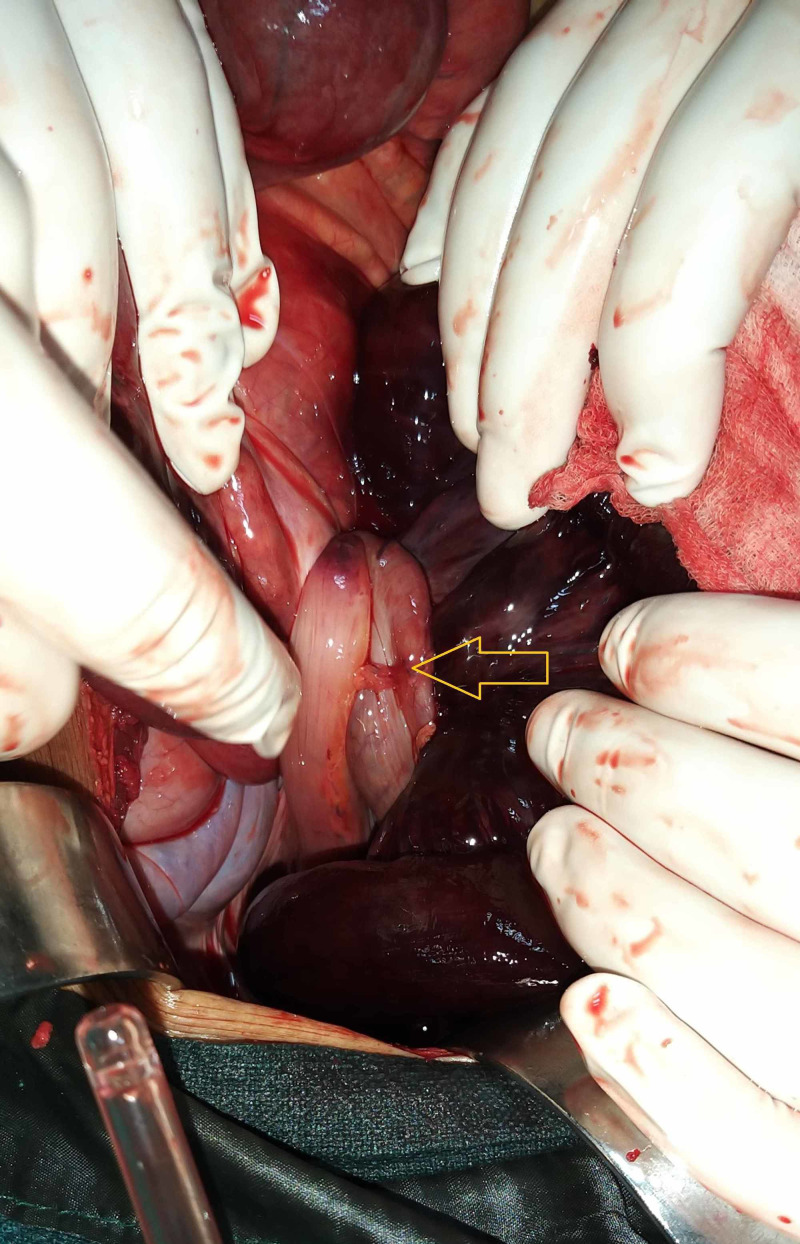
Intraoperative image showing the ileosigmoid knot (yellow arrow).

**Figure 3:**
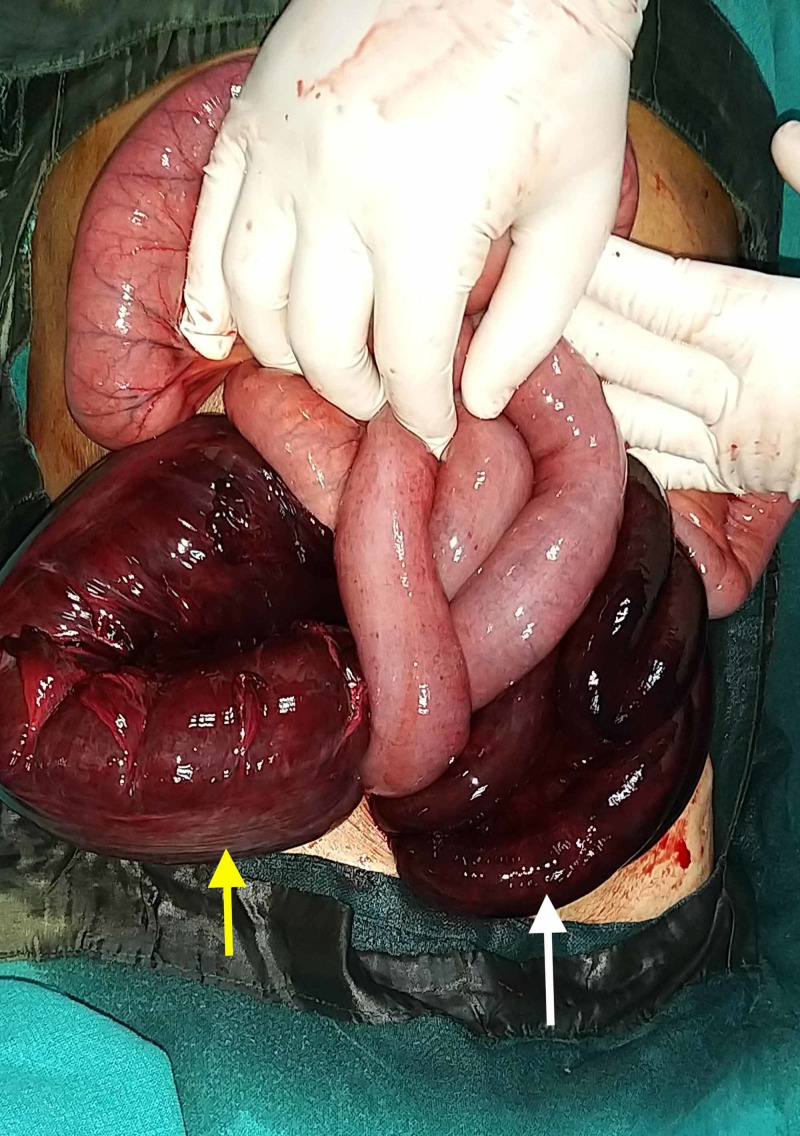
Intraoperative image showing gangrenous ileum (white arrow) and sigmoid colon (yellow arrow).

A 70-year-old male presented to the surgical emergency with complaints of abdominal pain for three days. The pain was insidious in onset, diffuse, non-radiating, and continuous. It was associated with a few episodes of bilious vomiting, obstipation, and abdominal distension. There was no history of fever, jaundice, or urinary complaints. The patient had no past surgical history but had a personal history of addiction to opium. On general examination, the patient was conscious, oriented, and afebrile. His pulse rate was 100 beats per minute, respiratory rate was 20 breaths per minute, and blood pressure was 100/50 mm of Hg. Examination of the abdomen revealed a distended abdomen with diffuse tenderness, guarding, rigidity, and evidence of free fluid. There was no obliteration of liver dullness, and bowel sounds were absent. The digital rectal examination revealed an empty rectum with ballooning. X-ray of the abdomen showed distended large bowel and multiple air-fluid levels in the small bowel with no free air under the right hemidiaphragm (Figure 1). The arterial blood gas analysis revealed features of metabolic acidosis. CT scan could not be performed in this case as the patient was having features of peritonitis. After initial resuscitation, emergency laparotomy was planned in view of features of acute intestinal obstruction with peritonitis. On exploration, there was a 360-degree twisting of the ileal loops around the sigmoid colon and its mesentery (Figure 2). There was gangrene of distal ileum approximately 100 cm in length, starting 6 cm proximal to the ileocolic junction along with gangrene of the sigmoid colon (Figure 3). Additionally, the mesentery of the sigmoid colon was long with narrow attachment and loaded with stool. The gangrenous bowel loops were resected, and an end ileostomy and descending end colostomy were performed in view of late presentation, old age, bowel edema, and peritonitis. The patient was discharged on postoperative day six with good general condition.

## Discussion

ISK is one of the uncommon but potentially life-threatening causes of bowel obstruction, in which a portion of the ileum and sigmoid colon wrap around each other, forming a closed-loop obstruction. It is commonly seen in the Africa, Asia, and the Middle East populations, with a male preponderance and mean age of presentation around 40 years [2,3]. Endemicity of the disease has also been reported in Turkey, specifically in Eastern Anatolia. It has been postulated that ISK is more commonly seen in redundant sigmoid colon with a narrow pedicle, ingestion of a high bulk diet in the presence of an empty small bowel, and a freely mobile small bowel with long mesentery. Hence, it has been seen commonly in Muslims who eat a heavy meal daily during the Ramzan fasting. Late pregnancy, transmesenteric herniation, Meckel diverticulitis with a band, ileocecal intussusception, and floating cecum are other secondary causative factors [4,5]. The common modes of presentations are colicky abdominal pain with obstipation, asymmetrical abdomen distension, and vomiting. Other associated clinical features are akinetic or hypokinetic bowel sounds, empty rectal vault on digital rectal examination, and hyperkinetic bowel sounds. Also, there can be rebound tenderness and rigidity on abdominal examination, suggesting peritonitis due to perforation. In gangrenous bowel, there may be melanotic rectal stool on per rectal examination. ISK is usually associated with toxic shock or hypovolemic shock and has been reported in 36.4% to 78.1% of patients. Erect X-ray of the abdomen typically reveals dilated sigmoid colon present on the right side and dilated small bowels with multiple air-fluid levels present on the left side [6]. Abdominal CT can reveal characteristic whirl sign. The findings of an ISK are not easily detected because the ileal twist is higher within the abdomen than the usual situation of a sigmoid volvulus [7]. The initial management requires rapid and effective resuscitation with intravenous fluids, correction of electrolytes and acid-base imbalance, and nasogastric tube decompression followed by an emergency laparotomy. Appropriate antibiotics are given in the perioperative period, usually a combination of cephalosporins, aminoglycosides, and metronidazole [1]. The operative procedure is decided based on the intraoperative findings and physiological status of the patient. In case of both loops are viable, the knot may be untwisted after sigmoid enterotomy. If both ileum and the sigmoid colon are gangrenous, the gangrenous part should be resected after applying the bowel clamp because of difficulty in untying the knot, and rupture of the gangrenous loop could lead to spillage of toxic bowel contents [4,8]. Although primary anastomosis of the small bowel is preferred, there are high chances of a leak if end-to-end anastomosis is performed in cases where the gangrenous ileum is within 10 cm from the ileocecal valve [5]. Sigmoid colectomy is usually performed in all instances irrespective of viability because of the risk of recurrent volvulus or repeat knotting due to the redundancy of the loop. The primary colonic anastomosis can be performed in case of early presentation, and healthy and undistended bowels. Hartmann’s procedure is considered suitable if the distal segment has doubtful viability and difficult to exteriorize without undue tension. The reported mortality from ISK varies from 0% to 48% [5]. Higher mortality has been observed in patients with old age (<60 years), delayed presentation (>24 hours), and the presence of gangrenous bowel [3]. Considering the presence of the above high-risk factors in our patient along with bowel edema and luminal discrepancy, resection of the gangrenous segments with end ileostomy and Hartmann’s procedure was performed in this case.

## Conclusions

ISK is a rare and life-threatening condition of closed-loop obstruction. It is sporadic and with high morbidity and mortality if it occurs in old age. Diagnosis of this disease is often difficult due to the rarity of the disease and variation in clinical presentation. Initial resuscitation and prompt diagnosis followed by early surgical intervention are vital in determining the morbidity and mortality of this disease. The surgical procedure performed is based on the patient’s physiological status and intraoperative findings. Resection of gangrenous bowel and restoration of the continuity should be the primary aim, but stoma can be created in the presence of high-risk factors to prevent undue mortality.
